# Expression of IL-35 in the Prophase of Liver Failure and its Immune Regulatory Interaction with Glucocorticoids

**DOI:** 10.2174/0118715303457167260506214057

**Published:** 2026-07-01

**Authors:** Jing Gu, Yan Wang, Xin-Chi Zhang, Yu-Fan Xiong, Xin-Yi Li, Tian-Dan Xiang, Li Chen, Jian-He Gan

**Affiliations:** 1 Department of Infectious Diseases, The First Affiliated Hospital of Soochow University, Suzhou, 215000, Jiangsu, China

**Keywords:** IL-35, glucocorticoids, liver failure, Treg, hepatitis B virus, Th17 cells

## Abstract

**Introduction:**

To investigate the immunoregulatory role of interleukin-35 (IL-35) in the prophase of liver failure related to hepatitis B virus (HBV-PLF) and its synergistic mechanism with glucocorticoid treatment.

**Methods:**

22 patients with HBV-PLF (matched with 22 healthy controls) were included. Peripheral blood regulatory T cells (Treg)/helper T cells 17 (Th17) ratio, serum IL-35/IL-17 dynamic changes, and liver function indicators were measured both before and after methylprednisolone treatment (1 mg/kg, 7 days). A concanavalin A-induced PLF mouse model was used to evaluate the effects of IL-35 overexpression, IL-35 neutralizing antibody, and dexamethasone on liver injury, cytokines, and the hepatic immune microenvironment.

**Results:**

Compared with healthy controls, patients with HBV-PLF exhibited a significantly reduced Treg/Th17 ratio and elevated serum IL-35 levels at baseline (*p* < 0.05). Following methylprednisolone treatment, both serum IL-35 levels and the Treg/Th17 ratio increased, with IL-35 levels showing a strong positive correlation with the Treg/Th17 ratio (*r* = 0.69). In the mouse model, IL-35 overexpression significantly alleviated liver injury, showing a hepatoprotective effect comparable to that of dexamethasone (*p* > 0.05). This improvement was characterized by decreased alanine aminotransferase levels and improved histopathological scores (*p* < 0.05). In contrast, administration of an IL-35 neutralizing antibody was found to exacerbate damage. Mechanistic investigations revealed that IL-35 promoted the proportion of hepatic FOXP3+ Treg cells while inhibiting Th17 activation, thereby establishing the Treg/Th17 balance.

**Discussion:**

This study demonstrates the dynamic immunoregulatory role of IL-35 in PLF through clinical observations and mechanistic validation in a mouse model. IL-35 was closely associated with restoration of the Treg/Th17 balance and showed hepatoprotective effects comparable to glucocorticoids, suggesting its potential as a target for early immunomodulatory intervention.

**Conclusion:**

IL-35 serves as a critical regulator of immune homeostasis in PLF. Glucocorticoids appear to exert their protective effects by upregulating IL-35 expression. Consequently, targeting the IL-35 pathway represents a promising strategy for early intervention in liver failure.

## 1 INTRODUCTION

Liver failure is a severe clinical syndrome characterized by extensive hepatocellular degeneration and necrosis resulting from diverse etiologies, including viral infections, drugs, and toxins, ultimately leading to decompensation of essential hepatic functions such as synthesis, detoxification, and metabolism. The treatment of liver failure is extremely challenging [1, 2]. Accumulating evidence indicates that dysregulated and excessive immune activation plays a central role in the pathogenesis of liver failure [3]. In the early phase following disease onset, the liver rapidly produces large amounts of pro-inflammatory cytokines, including tumor necrosis factor-α (TNF-α), interleukin-6 (IL-6), and interleukin-1β (IL-1β), thereby initiating a cytokine-mediated “inflammatory storm” [4, 5]. This excessive inflammatory response subsequently leads to systemic inflammatory response syndrome and may progress to multiorgan failure [6, 7].As the disease progresses, it may transition to a state of “immune paralysis”, characterized by immune suppression and an increased susceptibility to infections. Therefore, immunoregulatory treatment in the prophase of liver failure (PLF) is critical to effectively prevent its progression to liver failure [8].

Immunological dysregulation in the PLF is primarily characterized by the excessive activation of cellular immune responses [9]. During this phase, effector T lymphocytes undergo aberrant activation, and Th17 cells release high levels of IL-17, IL-6 and TNF-α that drive hepatic injury [10, 11]. Concomitantly, compensatory pathways drive a pronounced increase in regulatory T (Treg) cell numbers [12]. Treg cells inhibit the inflammatory response by secreting IL-10 and TGF-β. However, because of the difficulty in maintaining the balance between antiviral immunity and immunopathological damage, the Th17/Treg cell ratio becomes dysregulated, ultimately leading to uncontrolled immune responses and massive hepatocyte necrosis. Therefore, timely treatment of patients in the PLF and effective suppression of the excessively activated immune response can greatly help reduce the incidence of liver failure. Recent studies have shown that IL-35, a cytokine secreted by Treg cells, plays a central role in maintaining immune homeostasis [13]. IL-35 not only directly inhibits the activation of Th17 cells and promotes the secretion of anti-inflammatory cytokines such as IL-10 and TGF-β, but also induces the differentiation of a novel regulatory T cell (iTr35), resulting in the formation of a negative feedback regulatory loop [14]. Notably, in our previous studies, we found that the expression level of IL-35 is closely related to the prognosis of liver disease—excessively high levels may lead to immune suppression and secondary infections, while too low levels fail to effectively control inflammation [15, 16].

Glucocorticoids (such as methylprednisolone) are the main immunomodulatory agents used in the clinical treatment of PLF. However, their exact mechanism of action has not yet been fully elucidated [17]. Recent studies suggest that glucocorticoids may enhance immune suppression by upregulating the secretion of Treg cells [18]. Thus, we propose the following core hypothesis: In PLF, glucocorticoids alleviate liver injury by promoting IL-35 expression and restoring the Treg/Th17 balance. However, the dynamic changes that IL-35 undergoes in PLF and its synergistic mechanism with glucocorticoids remain unclear.

To verify this hypothesis, we employ a “clinical-basic” dual strategy. Clinically, serum levels of IL-35 and IL-17, and peripheral Treg/Th17 ratios were measured in HBV-related PLF patients before and after glucocorticoid treatment, and their correlation with hepatic function improvement was assessed. Mechanistically, the IL-35-mediated immune regulatory network is revealed by using a ConA-induced PLF mouse model, along with IL-35 overexpression and neutralizing antibody intervention.

## 2 MATERIALS AND METHODS

### 2.1 Reagents

Key reagents used in this study included concanavalin A (Con A; Sigma-Aldrich, St. Louis, MO, USA), dexamethasone (Sigma-Aldrich), and cytokine ELISA kits (USCN Life Science Inc., Wuhan, China). Primary antibodies against IL-12A and IL-17A were obtained from Abcam (Cambridge, UK), and the FOXP3 antibody was obtained from Affinity Biosciences (Jiangsu, China). The IL-35 expression plasmid was kindly provided by Dr. Yuejun Liu from the First Affiliated Hospital of Soochow University. Detailed information for all reagents, antibodies, and catalogue numbers is provided in Supplementary Table **S1**.

### 2.2 Clinical Study

#### 2.2.1 Study Population

A prospective observational study design was adopted, and 22 patients with HBV-related PLF treated from January 2018 to December 2019 were enrolled. These 22 patients were matched with 22 healthy controls. No statistical differences were found between the patient group and the healthy control group in terms of age (45.28±17.63 *vs* 48.41±8.42 years) and sex (*p* > 0.05).

#### 2.2.2 Inclusion and Exclusion Criteria

The included population should meet the following PLF diagnostic criteria as mentioned in the *Liver Failure Diagnosis and Treatment Guidelines* (2018 edition):

- Clinical symptoms: severe fatigue + gastrointestinal symptoms (anorexia/vomiting/abdominal distention);
- Laboratory indicators:
  - Total bilirubin (TBIL) 51–171 μmol/L, with a daily increase of >17 μmol/L.
  - 40% ≤ PTA ≤ 50% (INR < 1.5).
  - Significant elevation in ALT (alanine aminotransferase)/AST (aspartate aminotransferase).
- HBV infection: positive for hepatitis B surface antigen for more than 6 months.

The exclusion criteria were as follows: Infections caused by other hepatotropic viruses (hepatitis A, C, D, and E); alcoholic liver disease and autoimmune liver diseases; and comorbidities with other major diseases.

The study involving human participants was approved by the IRB of The First Affiliated Hospital of Soochow University (No. 2021-175). All participants signed informed consent forms.

#### 2.2.3 Glucocorticoid Administration Protocol for Patients

On the day of admission, patients with HBV-related PLF underwent routine biochemical and coagulation function tests and other relevant exclusionary diagnostic examinations. After excluding contraindications to glucocorticoid use and obtaining informed consent, the patients were administered methylprednisolone sodium succinate through injection at a dose of 1 mg/kg of body weight once daily. The dose was decreased by 10 mg every 5 days. Symptomatic supportive treatments, including gastric protection, liver protection, and anti-hepatitis B virus therapy, were simultaneously provided.

#### 2.2.4 Blood Sample Collection Time Points

Blood samples were collected for testing at baseline before beginning glucocorticoid treatment and after 7 days of treatment. Routine tests for blood counts, biochemistry, and coagulation were performed. Flow cytometry was used to analyze the classification and proportion of peripheral blood lymphocytes. Serum samples were also collected and stored at -80 °C for subsequent testing.

### 2.3 Animal Experiments

#### 2.3.1 Animals

Female BALB/c mice (8–12 weeks, 20–25 g) were supplied from Suzhou Zhaoyan New Drug Research Center Co., Ltd. (China). All animal experiments were approved by the IACUC of The First Affiliated Hospital of Soochow University (No.SUDA20201208A03) and performed in accordance with the National Research Council's Guide for the Care and Use of Laboratory Animals.

#### 2.3.2 Establishment of the PLF Mouse Model

Animals were randomly allocated to four intravenous ConA regimens (n = 15 each): a blank group, low-dose group (10 μg/kg), medium-dose group (20 μg/kg), and high-dose group (30 μg/kg). After 48 h of injection, the degree of liver damage in the mice was assessed by serum biochemical, pathological, and related inflammatory cytokine levels. Based on the pathology index (PI) score of liver tissue, the 20 μg/kg dose was selected as the optimal condition for subsequent experiments. Model mice were euthanised by cervical dislocation, bled *via* the orbital sinus, and the left hepatic lobe was removed for downstream assays. The remaining liver tissue was sliced into approximately 1 cm × 2-mm-thick sections. These liver tissue sections were then immersed in formalin solution and fixed for 48 h. The remaining liver tissue was snap-frozen at −80 °C for future studies.

#### 2.3.3 Experimental Grouping and Intervention

The experimental mice (n=75) were randomly divided into the PLF model group (called the model group), blank group, IL-35 plasmid group, anti-IL-35 antibody group, and dexamethasone group. Each group comprised 15 mice. All interventions were administered 24 h before PLF induction. PLF was induced in all groups except the blank group by tail vein injection of Con A (20 μg/kg, dissolved in 100 μL sterile saline). All mice were euthanised 48 h after Con A injection, and samples were collected as described above. The amplification method for the IL-35 plasmid and the specific intervention protocol are provided in Supplementary Figures **S1** and **S2**, and Table **S2**. All drug solutions were freshly prepared on the day of administration. Con A (jack bean protein), IL-35 plasmid, anti-IL-35 antibody, and dexamethasone were dissolved in sterile saline to achieve the required concentrations. The solutions were filtered through a 0.22 μm sterile filter before injection. Any unused solution was discarded and not reused. The entire experimental period, from the first intervention (24 h before modeling) to sample collection (48 h after modeling), lasted 72 hours for the main study.

### 2.4 Laboratory Tests

#### 2.4.1 ELISA Detection of IL-35, IL-17, IL-4, and TNF-α

Human serum samples were tested for IL-35 and IL-17 levels using the enzyme-linked immunosorbent assay (ELISA) method. The mouse serum was also tested by using the ELISA method. Specific antibodies were used to capture the corresponding cytokines. Substrate reactions were employed to generate color and determine the optical density (OD) values. The concentrations of each cytokine were calculated based on a standard curve. All experiments were performed according to the instructions mentioned on the human ELISA kit (Uscn Life Science, Wuhan, China) and the mouse ELISA kit (CusaBio, Wuhan, Hubei Province, China) to ensure the accuracy and reproducibility of the results obtained.

#### 2.4.2 Flow Cytometry Detection of Treg and Th17 Cells

Peripheral blood samples from patients were collected using anticoagulant tubes. Mononuclear cells were isolated through density gradient centrifugation on Ficoll–Paque. The isolated cells were surface-labelled for 20 min at room temperature in the dark with anti-CD4-PerCP, anti-CD127-FITC, and anti-CD25-APC (all from BD Bioscience, San Jose, CA, USA) antibodies. After two PBS washes to strip excess antibody, the cells were permeabilized and incubated with anti-IL-17-PE (BD Bioscience) for 30 min at 4 °C in the dark. Isotype-matched reagents served as gating controls for each fluorescence channel (PerCP, APC, FITC, and PE). Acquisition was performed on a FACS Calibur analyzer (BD Bioscience). Data were analysed with Cell Quest Pro software (BD Bioscience) and FlowJo software version 8.6 (Tree Star Inc., Ashland, OR, USA).

#### 2.4.3 HE Staining of Mouse Liver Tissue

Liver tissue samples were fixed in 10% neutral-buffered formalin. They were then processed and embedded in paraffin. Next, 5-µm-thick sections were cut and stained with H&E to observe histological changes in the liver tissue. Liver inflammation was assessed using the PI. The following scoring criteria were employed: “0”: no liver damage in the entire liver section; “1”: <1/3rd of the liver slice has damage; “2”: 1/3rd–2/3rd of the liver slice shows damage; and “3”: >2/3rd of the liver slice is damaged. Each section was assessed in two fields, and 20 fields per group were evaluated. The cumulative score gives the PI value that is used to determine the extent of liver damage.

#### 2.4.4 Immunohistochemistry and Immunofluorescence for IL-35, IL-17, and Treg

To assess inflammatory cytokines in liver tissue, paraffin-embedded liver tissue sections were incubated with primary antibodies to IL-12A, IL-17A (both Abcam, USA), and FOXP3 (Affinity, USA). A polymer-detection system (Zhongshan Golden Bridge Biotech, Beijing, China) enabled double immunohistochemical staining. A semi-quantitative scoring system was employed to assess histological changes by applying HRP-labeled broad-spectrum secondary antibodies (or fluorescent secondary antibodies) on the tissue slides and incubating them in a wet chamber. “Positive areas” refer to regions where the expression of target proteins (IL-35/IL-17/Foxp3) is significantly higher than that in the background following staining with specific antibodies, reflecting the spatial distribution of these proteins within the liver tissue.

### 2.5 Statistical Analysis

Continuous data are presented as mean ± standard deviation (SD), and categorical data are shown as *n* (%). Group differences were assessed using the rank-sum test and independent sample *t*-test, as necessary. Pearson correlation analysis and partial correlation analysis were performed to evaluate the relationships between the parameters. Two-sided *p*-values of less than 0.05 were considered statistically significant. All data analyses were performed using SPSS version 22.0 (SPSS Inc., Chicago, IL, USA), and graphical presentations were made using GraphPad Prism (version 9.0, California, USA).

## 3 RESULTS

### 3.1 Baseline Information of HBV-PLF Patients and Healthy Controls

Baseline characteristics are summarized in Table **1**. Mean age in the PLF group was 45.28±17.63 years, with 77% men, while the corresponding values for healthy controls were 48.41±8.42 years and 76.7% men, with no significant inter-group differences at baseline levels. After 1 week of methylprednisolone (1 mg/kg) treatment of the PLF group, 18 of 22 treated subjects showed clinical improvement, whereas four deteriorated to overt liver failure. Of these four, three improved after undergoing plasma exchange and symptomatic support, while the remaining patient required liver transplantation at another hospital because of the development of hepatic encephalopathy. Upon admission and 1 week after glucocorticoid treatment, TBIL, ALT, and AST decreased, whereas PTA increased after treatment than those of the pretreatment levels, indicating an improvement in liver function after glucocorticoid treatment. Hence, it can be suggested that glucocorticoids may help block further hepatocyte necrosis in patients with HBV-related liver failure.

### 3.2 Elevated IL-35 in Patients with HBV-PLF and Its Association with Immune Imbalance

Before glucocorticoid therapy, HBV-PLF patients exhibited significantly higher proportions of both Treg and Th17 cells compared to the control group. However, the increase in Th17 cells was more pronounced, leading to a significantly lower Treg/Th17 ratio in the PLF model group (Fig. **1A**). Similarly, before treatment, IL-35 and IL-17 cytokines in the PLF model group were significantly higher compared to the healthy group, indicating a severe cellular immune disorder in the patients (Fig. **1A**). Following one week of glucocorticoid administration, the proportion of Treg cells increased significantly, accompanied by a concomitant decrease in Th17 cells, effectively restoring the Treg/Th17 balance (Fig. **1B**). These findings suggest that glucocorticoid treatment partially corrected the cellular immune imbalance. Consistent with these cellular changes, serum analysis demonstrated increased IL-35 levels accompanied by decreased IL-17 levels (Fig. **1B**). The concordance between cytokine profiles and flow cytometric findings supports the potential role of glucocorticoid treatment in regulating the IL-35 and IL-17 levels in patients with HBV-PLF.

We further analyzed the correlation between IL-35 and IL-17 with the Treg/Th17 ratio (Fig. **2**). A strong positive correlation was observed between IL-35 levels and the Treg/Th17 ratio (*r* = 0.69), whereas IL-17 levels showed no significant correlation with the Treg/Th17 ratio. These findings suggest that IL-35 may exert its immunoregulatory effect by modulating the Treg/Th17 balance.

### 3.3 Protective Role of IL-35 in Liver Failure in the Mouse Model

#### 3.3.1 Construction and Validation of the ConA-Induced PLF Model

BALB/c mice were used to establish an immune-induced liver injury model by tail-vein injection of concanavalin A (ConA). Mice were allocated to different dose groups (n = 15 per group). The severity of liver injury was dose-dependent. During the experimental period, mortality was observed primarily in the ConA-induced PLF model. In the main experimental cohort, 4–5 mice died in the model group before the scheduled endpoint (48 h after ConA injection), corresponding to a mortality rate of approximately 26–33%. Most deaths occurred within the first 24–36 h after ConA administration, consistent with the acute inflammatory phase of immune-mediated liver injury. No deaths were observed in the control group. Serum biochemical analyses demonstrated that total bilirubin, AST, and ALT levels were gradually increased with escalating ConA doses (*p* < 0.05). Histopathological examination showed clear hepatocyte damage and local lymphocyte infiltration, consistent with the pathological changes seen in immune-mediated liver injury. In the low-dose group (10 μg/kg), hepatocytes exhibited mild swelling with small areas of necrosis; in the medium-dose group (20 μg/kg), focal hepatocyte necrosis was observed; and in the high-dose group (30 μg/kg), large areas of hepatocyte necrosis were noted (Figure **S3**). Serum levels of IL-17, IL-4, and TNF-α increased with the ConA dose (Supplemetary Table **S3**). Given that ConA at 20 μg/kg induced liver injury with moderate inflammation and reversible necrosis, resembling the pathological features of PLF, this dose was selected for subsequent experiments.

#### 3.3.2 IL-35 Intervention Alleviates Liver Injury

After establishing the ConA-induced mouse liver failure model, we evaluated the therapeutic effects of IL-35. Forty-eight hours after model induction, serum liver function markers, including TBIL, ALT, and AST, were significantly elevated compared with the control group (*p* < 0.05), confirming the successful establishment of liver injury (Figs. **3A**-**C**). Administration of the IL-35 plasmid yielded robust hepatoprotection, as evidenced by significantly reduced TBIL, ALT, and AST levels compared with those in the model group (*p* < 0.05, Figs. **3A**-**C**). The extent of improvement was comparable to that achieved with dexamethasone, with no statistically significant difference between the two treatments (*p* > 0.05). Conversely, administration of the anti-IL-35 antibody exacerbated hepatic dysfunction, producing significantly higher levels of these markers than the model group (*p* < 0.05, Figs. **3A**-**C**), underscoring the critical protective role of endogenous IL-35. Collectively, these findings indicate that IL-35 can exert glucocorticoid-comparable hepatoprotection *via* its unique immunoregulatory mechanisms. Histological examination further supported these findings. Mice receiving either the IL-35 plasmid or dexamethasone exhibited only mild hepatocyte edema and focal inflammatory infiltration, with mean histopathological scores of 2.11 ± 0.65 and 2.15 ± 0.96, respectively, significantly lower than those of the model group (2.91±0.72, *p* < 0.05, Figs. **3D** and **4**). Notably, the anti-IL-35 antibody group exhibited aggravated liver injury, including more extensive necrotic areas and more significant inflammatory infiltration (pathological score of 3.75±1.28, *p* < 0.05, Figs. **3D** and **4**). Quantitative analysis revealed that histological improvement in IL-35–treated mice was comparable to that observed with dexamethasone (*p* > 0.05). These pathological findings were highly consistent with the serum biochemical changes, further confirming the protective effect of IL-35 against liver injury.

#### 3.3.3 IL-35 Regulates the Local Immune Microenvironment

To explore the hepatoprotective mechanism of IL-35, we further analyzed changes in the local immune microenvironment of the liver (Table **2**). In PLF model mice, the expression of pro-inflammatory cytokines, including IL-17, IL-4, and TNF-α, was significantly increased compared with blank controls. Interestingly, the anti-inflammatory cytokine IL-35 was also significantly increased (Fig. **5**). Administration of either the IL-35 plasmid or dexamethasone substantially reduced pro-inflammatory cytokine levels compared with untreated PLF mice, accompanied by a further increase in serum IL-35 (Fig. **5**). Similarly, anti-IL-35 antibody intervention blocked this protective effect. Mice in the anti-IL-35 group showed significantly higher levels of IL-4, IL-17, and TNF-α, supporting that endogenous IL-35 acts as a key regulator in the pathogenesis of PLF (cytokine storm) and is closely related to liver injury.

Next, we performed immunohistochemical analysis. The results showed that the positive cells were clearly structured, with IL-12a (a component of IL-35) and IL-17 appearing as distinct brown–yellow deposits that were clearly stronger than background staining (Fig. **6A**-**J**). Further immunofluorescence analysis revealed higher IL-35 expression levels (red fluorescence) in Foxp3^+^ Treg cells (green fluorescence), indicating that Treg cells are likely a major source of IL-35 in this setting (Fig. **6**). Together, these findings support a model in which IL-35 may exert its immunoregulatory effect through the “Treg–IL-35–Th17” axis.

Further quantitative immunohistochemical analysis revealed that, compared to the control group, liver tissues from the PLF model group displayed significantly greater positive areas for IL-17 and IL-35 (Table **3** and Fig. **7**, *p* < 0.05). However, among the treatment groups, mice receiving the IL-35 plasmid or dexamethasone exhibited significantly reduced IL-17-positive areas and significantly increased IL-35-positive areas compared with the model group (*p* < 0.05). Conversely, the anti-IL-35 antibody group exhibited a further increase in IL-17-positive areas and a decrease in IL-35-positive areas. Notably, treatment with the IL-35 plasmid was more effective than dexamethasone in decreasing IL-17-positive areas (*p* < 0.05) and in increasing IL-35-positive areas (*p* < 0.05). However, serum IL-35 levels measured by ELISA showed no significant difference between these two treatment groups (*p* > 0.05), suggesting that changes in the IL-35 expression in local tissues may be independent of systemic circulating levels (Fig. **7**).

## 4 DISCUSSION

This study provides new insight into the immunoregulatory mechanisms involved in the prophase of HBV-related liver failure (HBV-PLF). By integrating clinical observations with mechanistic validation in a ConA-induced mouse model, we demonstrated that IL-35 is dynamically associated with immune imbalance during PLF and may participate in the immunomodulatory effects of glucocorticoids. In China, hepatitis B virus (HBV) represents the predominant etiology of severe hepatitis. Immune disorders and hepatocyte necrosis are key mechanisms leading to HBV-related liver failure [19-21]. This study, integrating clinical cohorts and mouse models, is the first to systematically reveal the dynamic immunoregulatory role of IL-35 in HBV-related PLF. Clinical data showed that patients with HBV-PLF exhibited a marked immune imbalance characterized by a reduced Treg/Th17 ratio and elevated circulating IL-35 levels. These findings suggest that although compensatory anti-inflammatory pathways are activated during PLF, they may be insufficient to counteract excessive inflammatory activation. However, after one week of methylprednisolone treatment, liver function improved, and the Treg/Th17 immune imbalance was also partially restored. This coordinated change supports a potential link between glucocorticoid-mediated immune modulation and IL-35-associated regulatory pathways.

In the experimental model, IL-35 overexpression significantly attenuated ConA-induced liver injury and produced hepatoprotective effects comparable to those of dexamethasone. Conversely, neutralization of IL-35 aggravated liver injury, highlighting the protective role of endogenous IL-35 in immune-mediated hepatic inflammation. Mechanistically, IL-35 intervention promoted the proportion of hepatic FOXP3+ regulatory T cells while suppressing Th17 activation and pro-inflammatory cytokine production. These observations support the existence of a “Treg–IL-35–Th17” regulatory axis that may play a key role in maintaining immune homeostasis during the early phase of liver failure. Previous studies have reported that IL-35 is primarily secreted by regulatory T cells and can suppress effector T-cell responses while promoting regulatory immune pathways [22, 23]. Through inhibition of Th17 differentiation and enhancement of Treg stability, IL-35 contributes to the resolution of inflammatory responses in multiple immune-mediated diseases [24-26]. The present findings are consistent with these reports and further extend the immunoregulatory role of IL-35 to the context of HBV-related liver failure [27-29].

Another important observation of this study is the potential interaction between glucocorticoid therapy and IL-35-mediated immune regulation. Glucocorticoids are widely used immunomodulatory agents in the treatment of severe hepatitis and PLF. Previous studies have shown that glucocorticoids can regulate the balance between regulatory and effector T cells by enhancing FOXP3 expression and promoting the expansion of Treg cells [30-32]. In line with these findings, glucocorticoids may stimulate IL-35 production by increasing Treg activity [33]. Our clinical observations that IL-35 levels increased following methylprednisolone treatment provide further support for this hypothesis. Interestingly, immunohistochemical analyses in our study indicated that IL-35 expression within liver tissue was more pronounced in the IL-35 plasmid group than in the dexamethasone group, whereas circulating IL-35 levels were comparable between the two treatments. This discrepancy suggests that intrahepatic immune regulation may not be fully reflected by systemic cytokine levels and highlights the importance of evaluating local immune microenvironments in liver disease.

From a clinical perspective, these findings may have important implications for the management of severe hepatitis and early liver failure. First, IL-35 may serve as a potential immunological biomarker reflecting disease progression and immune status during PLF. In our cohort, baseline IL-35 levels were significantly lower in patients who subsequently progressed to overt liver failure compared with those who responded favorably to treatment. This observation suggests that insufficient IL-35-mediated immune regulation may contribute to uncontrolled inflammation and disease progression. Previous studies have also reported that circulating IL-35 levels are associated with disease severity in chronic viral hepatitis and acute-on-chronic liver failure [28, 34-36]. For example, plasma IL-35 levels were significantly associated with prognosis in patients with HBV-related acute-on-chronic liver failure and improved predictive performance when combined with the MELD score [6]. Similarly, studies in chronic hepatitis B and hepatitis C have demonstrated that IL-35 levels correlate with inflammatory activity and disease severity, suggesting that IL-35 may reflect the dynamic balance between antiviral immunity and immune-mediated liver injury. In addition, IL-35 has been reported to function as an immunoregulatory biomarker in several immune-mediated diseases by modulating the Treg/Th17 balance and suppressing excessive inflammatory responses. Therefore, dynamic monitoring of IL-35 may help identify early immunological deterioration and guide individualized immunomodulatory therapy. In addition, targeting the IL-35 signaling pathway may represent a novel therapeutic strategy for restoring immune homeostasis during the early stages of liver failure [15, 24, 36]. The proposed mechanism is summarized in Fig. (**8**).

## STUDY LIMITATIONS

This study has some limitations, too. Firstly, the clinical sample size was small (*n* = 22). In addition, this study was conducted at a single center. Thus, future large-sample, multicenter validation studies are needed. Secondly, although the ConA model simulates immune-mediated liver injury, it differs from the immune-initiation mechanism of viral liver failure [37, 38]. Thirdly, this study did not fully explore the effects of IL-35 on other immune cells, such as Kupffer cells and NK cells.

## CONCLUSION

This study confirms that IL-35 is a key regulatory factor in immune homeostasis in the PLF and acts through “bidirectional regulation” of the Treg/Th17 balance to alleviate liver injury. This finding not only deepens the current understanding of the immunopathology of liver failure but also provides a theoretical basis for the development of precision immunotherapies based on IL-35. Thus, targeting the IL-35 pathway may offer a new strategy for early intervention in liver failure.

## Figures and Tables

**Fig. (1):**
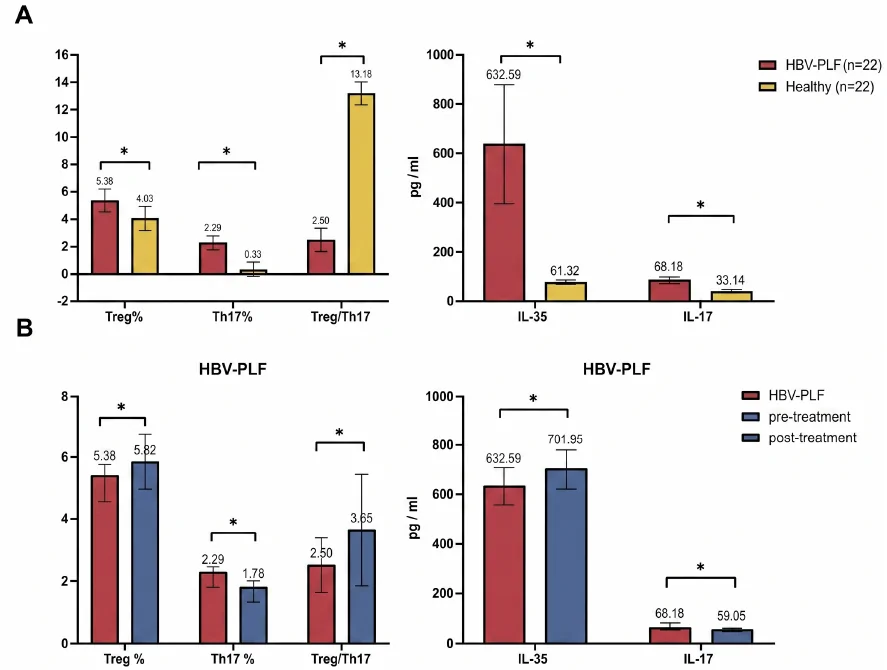
Immune cell subset distribution and cytokine levels in patients with HBV-PLF and healthy controls before and after treatment. (**A**) Comparison of the percentages of Treg, Th17, and the Treg/Th17 ratio between patients with HBV-PLF and healthy controls. Serum levels of IL-35 and IL-17 are also shown. (**B**) In the HBV-PLF group, changes in Treg, Th17, and Treg/Th17 ratio, along with IL-35 and IL-17 serum levels before and after treatment, are shown. *p < 0.05 for all comparisons marked by asterisks.

**Fig. (2):**
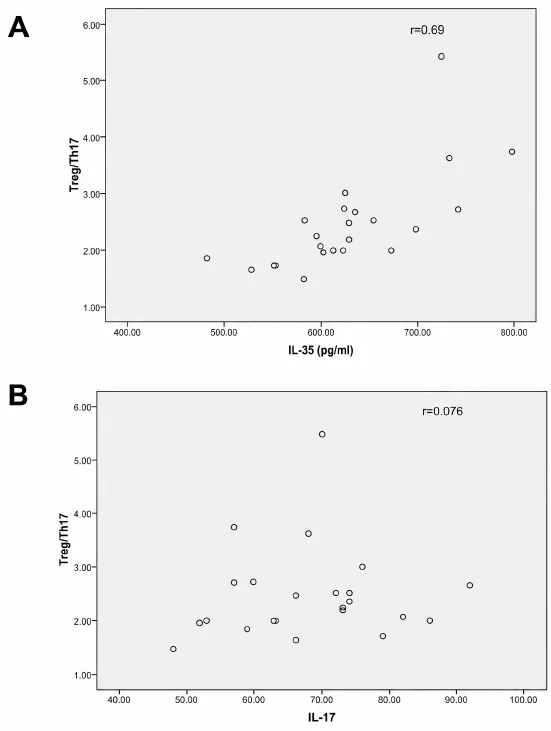
Correlation between serum IL-35/IL-17 and Treg/Th17 ratio in patients with HBV-PLF before treatment.

**Fig. (3):**
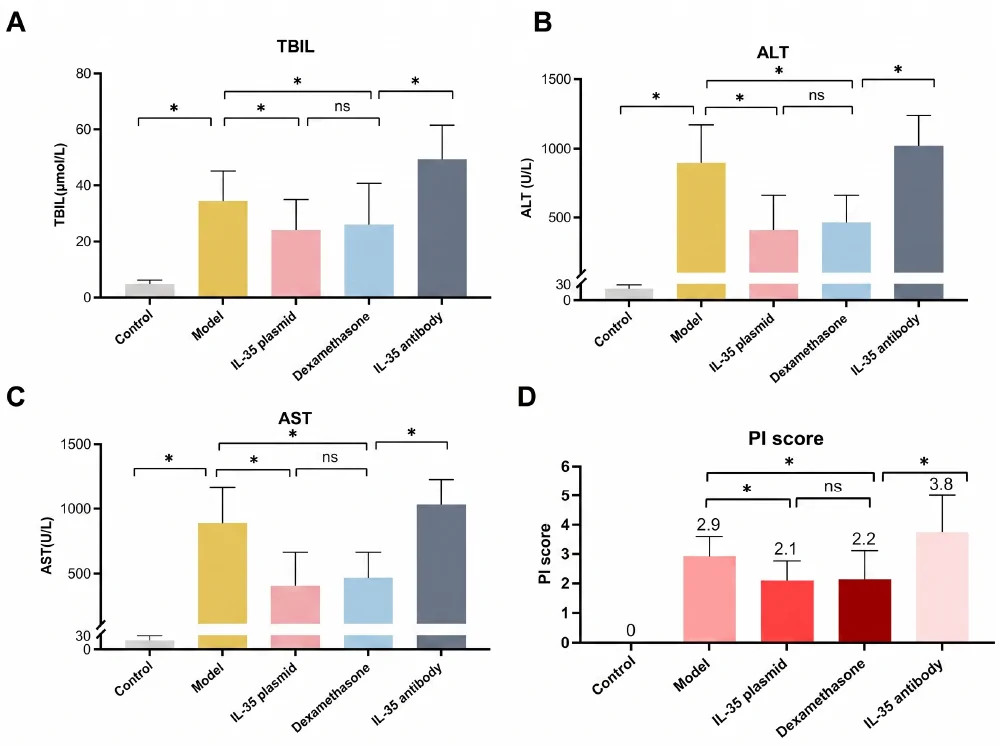
Effects of IL-35 plasmid, dexamethasone, and anti-IL-35 antibody treatments on liver function and pathological scores in a mouse model of liver injury. (**A**) Serum total bilirubin (TBIL) levels in the control, model, IL-35 plasmid, dexamethasone, and anti-IL-35 antibody groups. (**B**) Serum alanine aminotransferase (ALT) levels in each group. (**C**) Serum aspartate aminotransferase (AST) levels in each group. (**D**) Pathological index (PI) scores in each group. The sample size for each group of mice was 15. *p < 0.05 for comparisons marked by asterisks; ns indicates no significant difference.

**Fig. (4):**
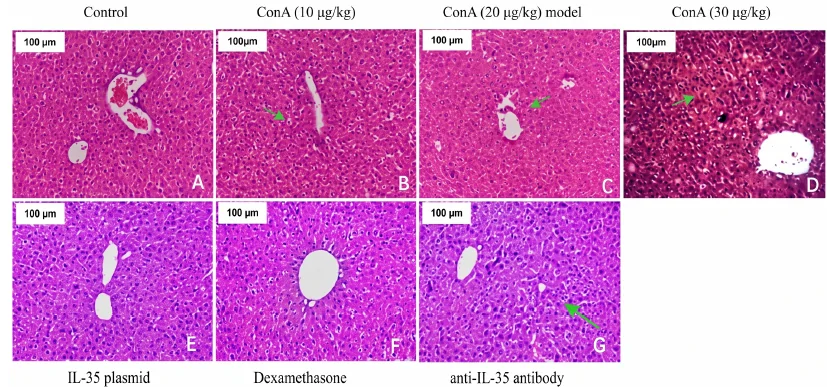
H&E staining of liver tissues in mice from the model, IL-35 plasmid, dexamethasone, and anti-IL-35 antibody groups. (**A**) Control group showing normal hepatic architecture. (**B**) ConA (10 μg/kg) group. The arrow indicates mild hepatocellular swelling in zone III of the hepatic acinus without obvious necrosis. (**C**) ConA (20 μg/kg) model group. The arrow indicates more pronounced hepatocellular swelling in zone III of the hepatic acinus. (**D**) ConA (30 μg/kg) group. The arrow indicates extensive hepatocellular necrosis in zone III of the hepatic acinus. (**E**) IL-35 plasmid group. (**F**) Dexamethasone group. (**G**) Anti-IL-35 antibody group. The sample size for each group was n = 15 mice. All scale bars represent 100 μm.

**Fig. (5):**
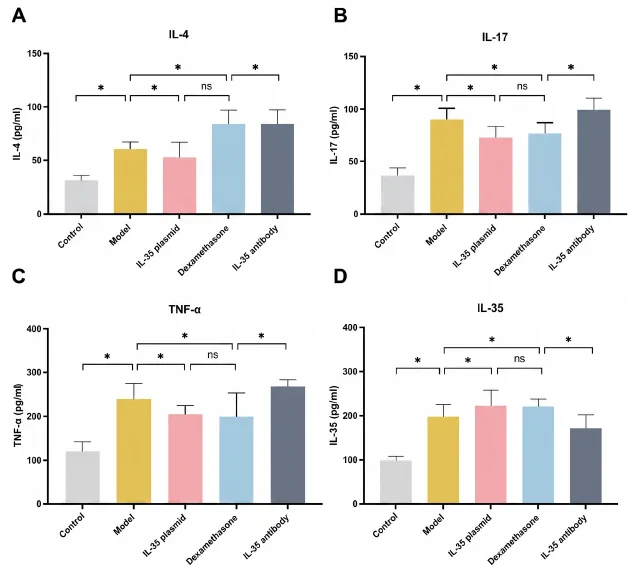
(**A**) Serum IL-4 levels in each group. (**B**) Serum IL-17 levels in each group. (**C**) Serum TNF-α levels in each group. (**D**) Serum IL-35 levels in each group. The sample size for each group of mice was 15. *p < 0.05 for all comparisons marked by asterisks; ns indicates no significant difference. Serum cytokine levels in the control, model, IL-35 plasmid, dexamethasone, and anti-IL-35 antibody groups.

**Fig. (6):**
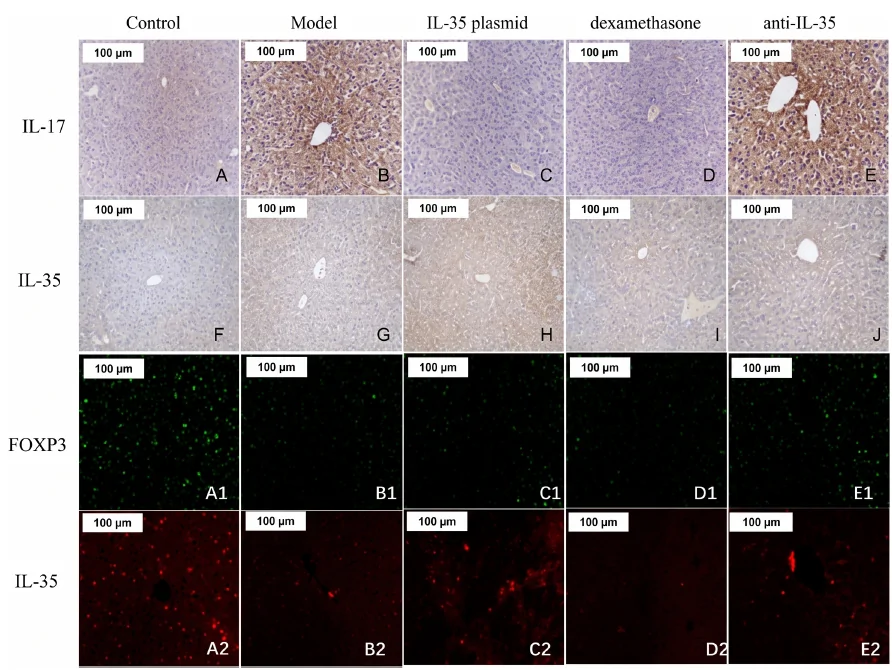
Immunohistochemical and fluorescent expression of IL-35 and FOXP3^+^ in liver tissues of mice from control, PLF model, and treatment groups. (**A-E**) IL-17 expression in liver tissues of the control group, PLF model group, IL-35 plasmid group, dexamethasone group, and anti-IL-35 antibody group, as assessed by immunohistochemistry. (**F–J**) IL-35 expression in liver tissues of the control group, PLF model group, IL-35 plasmid group, dexamethasone group, and anti-IL-35 antibody group, as assessed by immunohistochemistry. (A1–E1) Fluorescent levels of FOXP3**^+^** in liver tissues of the control group, PLF model group, IL-35 plasmid group, dexamethasone group, and anti-IL-35 antibody group. (A2–E2) Fluorescent levels of IL-35 in liver tissues of the control group, PLF model group, IL-35 plasmid group, dexamethasone group, and anti-IL-35 antibody group. All scale bars represent 100μm.

**Fig. (7):**
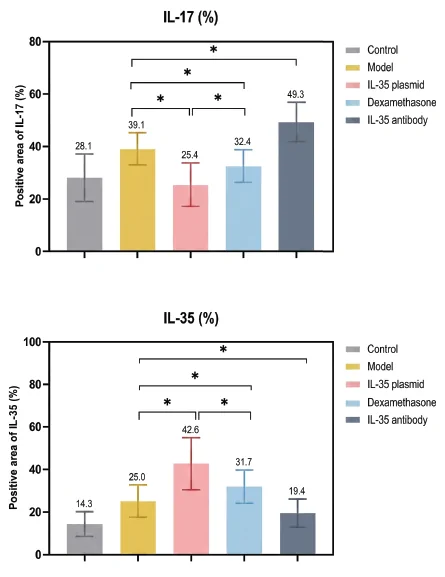
Percentage of positive areas for IL-17 and IL-35 in liver tissues of mice from control, model, and treatment groups. The sample size for each group of mice was 15. *p < 0.05 for all comparisons marked by asterisks.

**Fig. (8):**
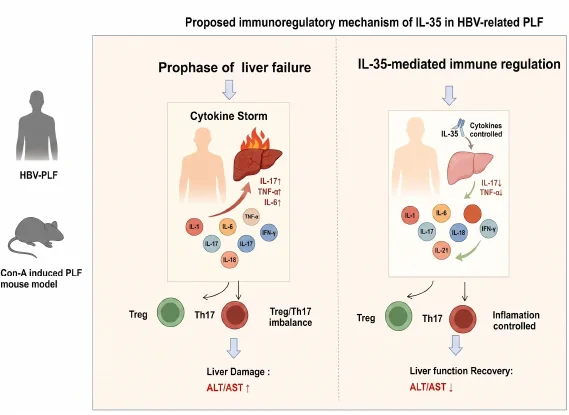
Proposed immunoregulatory mechanism of IL-35 in the prophase of HBV-related liver failure. During the prophase of liver failure, excessive inflammatory activation leads to a cytokine storm characterized by increased levels of pro-inflammatory cytokines such as IL-17, TNF-α, and IL-6, accompanied by an imbalance between regulatory T cells (Treg) and T helper 17 (Th17) cells. IL-35-mediated immune regulation restores the Treg/Th17 balance by enhancing Treg activity and suppressing Th17 responses, thereby reducing inflammatory cytokine production and alleviating liver injury.

**Table 1:** Baseline information of patients with prophase of liver failure before and after treatment.

**Index**	**HBV-PLF Group Before Treatment (*n* = 22)**	**HBV-PLF Group After Treatment (*n* = 22)**	**Control (*n* = 22)**
Male (%)	17 (77%) ^#^	17 (77%) ^#^	16 (76.7%) ^#^
Age (years)	45.28±17.63^#^	45.28±17.63^#^	48.41±8.42^#^
ALT (U/L)	2036±749**	1526±383**	25± 9
AST (U/L)	2219±892**	1392±401**	20±7
TBil (µmol/L)	138±37**	94±22**	12±5
PTA (%)	44.27±6.28**	51.82±11.21**	90±14
Cr (μmol/L)	52.24±19.31^#^	57.57±13.52^#^	56.9±15.82^#^
AFP (μg/L)	23.26±14.59*	19.45±4.73*	5.26±2.42*

**Abbreviations:** ALT, alanine aminotransferase; TBIL, total bilirubin; PTA, prothrombin time activity; AFP, alpha fetoprotein; Cr, creatinine. *Significant difference between the treatment group and the healthy control group before and after treatment (*p* < 0.05); #No significant difference between the treatment group before and after treatment compared with the healthy control group (*p* > 0.05); **Significant difference before and after glucocorticoid treatment (*p* < 0.05).

**Table 2:** Serum IL-4, IL-17, IL-35, and TNF-α in control group, model group, and each treatment group.

**Group (*n* = 15)**	**IL-4 (pg/mL)**	**IL-17 (pg/mL)**	**TNF-α (ng/L)**	**IL-35 (pg/mL)**
Control	31.17±4.96	36.24±7.71	120.18±22.57	100.39±10.54
Model group	60.21±7.04	89.74±10.52	241.14±33.41	199.78±26.15
IL-35 plasmid group	49.60±9.00	73.67±10.36	206.55±21.08	223.91±30.21
Dexamethasone group	52.33±13.13^#^	76.86±11.72^#^	200.27±15.21^#^	221.34±31.44^#^
anti-IL-35 antibody group	84.77±14.86	99.2±10.11	267.83±50.49	173.23±16.53
*p*-value	<0.05	<0.05	<0.05	<0.05

**Note:** ^#^No significant difference between IL-35 plasmid group and dexamethasone group (*p* > 0.05).

**Table 3:** Comparison of positive expression areas of IL-17 and IL-35 in liver tissue of mice in the control group, model group, and each treatment group.

**Group (*n* = 15)**	**IL-17-positive area (%)**	**IL-35-positive area (%)**
Control	28.07±8.96	14.26±5.86
Model group	39.12±6.13	24.95±7.48
IL-35 plasmid group	25.43±8.28	42.63±12.18
Anti-IL-35 antibody group	49.31±7.5	19.39±6.59
Dexamethasone group	32.43±6.28	31.67±7.81
*t1*	5.22*	4.79*
*t2*	3.46*	2.49*
*t3*	2.58*	2.28*
*t4*	2.45*	2.54*

**Note:** *t1*: IL-35 plasmid group *vs* model group; t2: anti-IL-35 antibody group *vs* model group; t3: dexamethasone group *vs* model group; t4: IL-35 plasmid group *vs* dexamethasone group; *p < 0.05.

## Data Availability

The data sets used and/or analysed during this study are available from the corresponding author upon request.

## LIST OF ABBREVIATIONS

HBV-PLF: The Prophase of Liver Failure Related to the Hepatitis B Virus
PLF: The Prophase of Liver Failure
Treg: Regulatory T Cells
Th17: Helper T Cells 17
ALT: Alanine Aminotransferase
AST: Aspartate Aminotransferase
IL-35: Interleukin-35
TBIL: Total Bilirubin
ELISA: Enzyme-Linked Immunosorbent Assay
OD: Optical Density
SD: Standard Deviation
ConA: Concanavalin A
HBV: Hepatitis B Virus
PTA: Prothrombin Time Activity
INR: International Normalized Ratio
TNF-α: Tumor Necrosis Factor-Alpha
IL-4: Interleukin-4
IL-6: Interleukin-6
IL-1β: Interleukin-1 Beta
IL-17: Interleukin-17
IL-10: Interleukin-10
IL-12a: Interleukin-12 Alpha
TGF-β: Transforming Growth Factor-Beta
PBS: Phosphate-Buffered Saline

## STANDARDS OF REPORTING

STROBE guidelines and methodology were followed.
