# Supplementary material for: Expression of IL-35 in the Prophase of Liver Failure and its Immune Regulatory Interaction with Glucocorticoids
Source: Endocr Metab Immune Disord Drug Targets. 2026 Jul 1;26:E18715303457167. doi: 10.2174/0118715303457167260506214057 (PMC13635969; doi:10.2174/0118715303457167260506214057)
Supplement: Supplementary file 1 [file EMIDDT-26-E18715303457167_SD1.pdf]

Supplementary Material

Expression Of IL-35 in the Prophase of Liver Failure and its Immune Regulatory Interaction with Glucocorticoids

Jing Gu[1,#] , Yan Wang[1,#] , Xin-Chi Zhang[1] , Yu-Fan Xiong[1] , Xin-Yi Li[1] , Tian-Dan Xiang[1] , Li Chen[1,*] and Jian-He Gan[1,*]

[1]*Department of Infectious Diseases, The First Affiliated Hospital of Soochow University, Suzhou, 215000, Jiangsu, China*

1 REAGENTS, ANTIBODIES AND MATERIALS USED IN THIS STUDY

Table S1. Reagents, antibodies and materials used in this study.

**Reagent / Material**	**Manufacturer**	**Catalogue No. / In-formation**
Paraffin	Shanghai Pharmaceutical Group, China	–
Formaldehyde	Shanghai Pharmaceutical Group, China	–
Xylene	Shanghai Pharmaceutical Group, China	–
Absolute ethanol	Shanghai Pharmaceutical Group, China	–
Ammonia solution	Shanghai Pharmaceutical Group, China	–
Hematoxylin	BASO Biotechnology Co., China	–
Eosin	BASO Biotechnology Co., China	–
Neutral resin mounting medium	Beijing Solarbio Science & Technology Co., China	–
Concanavalin A (Con A, Type IV)	Sigma-Aldrich, USA	–
Mouse IL-4 ELISA Kit	CusaBio, Wuhan, Hubei Province, China	–
Mouse IL-10 ELISA Kit	CusaBio, Wuhan, Hubei Province, China	–
Mouse IL-17 ELISA Kit	CusaBio, Wuhan, Hubei Province, China	–
Mouse TNF-α ELISA Kit	CusaBio, Wuhan, Hubei Province, China	–
Mouse IL-35 ELISA Kit	CusaBio, Wuhan, Hubei Province, China	–
Anti-β-Actin antibody (7G6)	CMCTAG	Batch No. 1832NJ
Anti-IL-12A antibody (EP5737)	Abcam, UK	–
Anti-IL-17A antibody (EPR21776)	Abcam, UK	–
Anti-FOXP3 antibody	Affinity Biosciences, China	DF6127
Anti-GITR antibody	Abcam, UK	–
Anti-GITRL antibody	Abcam, UK	–

Goat Anti-Rabbit IgG (H+L) HRP	CMCTAG	0822WB
Goat Anti-Mouse IgG (H+L) HRP	Bioworld Technology, USA	BS12478
pcDNA3.1 (+) expression vector	Invitrogen, USA	–
Escherichia coli DH5α	Laboratory stock	–
IL-35 plasmid	Gift from Dr. Liu Yuejun, The First Affiliated Hospital of Soochow University	–

2. IL-35 PLASMID AMPLIFICATION METHOD

2.1 Preparation of Competent Cells

Pick a single colony of the recipient strain (DH5α) grown on Luria-Bertani (LB) solid medium (provided by the laboratory), and inoculate it into 5 mL of LB liquid medium. Incubate the culture at 37°C with shaking overnight. Then, take 1 mL of the overnight culture and inoculate it into 100 mL of LB liquid medium, and incubate at 37°C with shaking at 300 rpm for about 1.5-3 hours, until the OD600 reaches 0.4-0.6. Under sterile conditions, transfer the bacteria into a pre-chilled sterile 50 mL centrifuge tube, and place it in an ice bath for 10 minutes. Centrifuge at 4°C at 8000 rpm for 10 minutes, discard the supernatant, and resuspend the pellet in 10 mL of ice-cold 0.1 mol/L $CaCl_2$. Incubate on ice for 10 minutes, then centrifuge at 4°C at 6000 rpm for 10 minutes and discard the supernatant. Repeat this step once. For every 50 mL of the initial culture, resuspend the bacterial pellet in 2 mL of pre-chilled 0.1 mol/L $CaCl_2$. Aliquot 200 µL into each Eppendorf tube, keep on ice for use within 12-24 hours, or add glycerol to a final concentration of 15% and store at -80°C for long-term use.

2.2 Transformation of Ligated Product

Take the prepared competent DH5α cells and thaw them slowly on ice. After 10 minutes in an ice bath, add 10 µL of the ligated product containing the IL-35 gene and the UMD 19-T-simple vector to 200 µL of the competent cells from the previous step. Gently rotate to mix the contents and place the tube on ice for 30 minutes. Heat shock at 42°C for 90 seconds, then quickly transfer the tube to an ice bath for 1-2 minutes. Add 800 µL of LB medium to each tube and incubate at 37°C with shaking for 45 minutes to allow the bacteria to recover. After centrifugation at 5000 rpm for 5 minutes, discard most of the supernatant under sterile conditions, leaving about 100 µL of culture medium. Resuspend the cells, and under the laminar flow hood, transfer the bacterial suspension onto an LB plate containing ampicillin, X-gal, and IPTG. Spread the suspension evenly, wait for the liquid to be absorbed, then invert the plate and incubate at 37°C for 12-16 hours.

2.3 Screening and Identification of Positive Clones

After transformation, perform blue/white colony screening. Pick the white colonies as positive clones, and extract the plasmid following the plasmid mini-prep kit instructions. The enzyme digestion electrophoresis identification system is as follows: 10 µL plasmid, 2 µL of 10× buffer, 1 µL of each Kpn I and EcoR I restriction endonuclease, 6 µL of distilled water, and incubate at 37°C in a water bath for 3 hours. After identifying the preliminary positive enzyme digestion results, store the corresponding plasmids for future use.

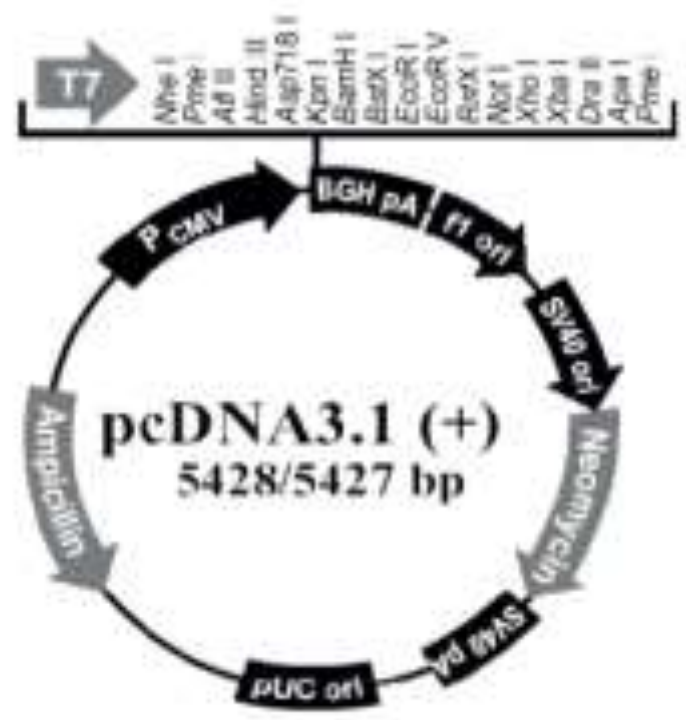

Figure S1. IL-35 plasmid structure diagram.

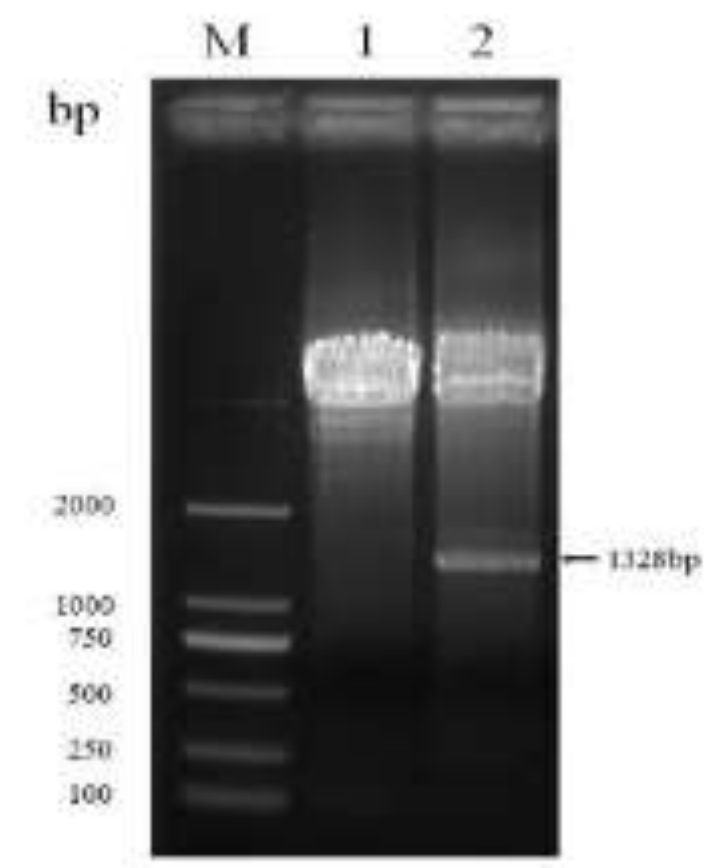

Figure S2. IL-35 digestion map.

3. SPECIFIC INTERVENTION PLAN FOR MICE

The specific intervention plan for mice in each group is outlined below:

Table S2: Specific Intervention Plan for Mice in Each Group.

**Group**	**Treatment Factor**
Blank group	100 µL physiological saline injected *via* tail vein
Model group	100 µL of jack bean protein solution injected *via* tail vein (dose: 20µg/kg)
IL-35 plasmid group	50ug IL-35 plasmid injected *via* tail vein 24 hours before modeling, dissolved in sterile saline to a final volume of 100 µL
IL-35 antibody group	400ug anti-IL-35 antibody injected intraperitoneally 24 hours before modeling, dissolved in sterile saline to a final volume of 100 µL
Dexamethasone group	3.6ug dexamethasone injected *via* tail vein 24 hours before modeling, dissolved in sterile saline to a final volume of 100 µL

3.1 Gross Specimens and HE Staining of Mice Livers in Various Doses of Jack Bean Protein Groups

Normal group: Under the microscope, liver cells appear normal in morphology, and the liver lobule structure is intact.

Low-dose group: A small amount of lymphocyte infiltration is observed around the central vein of the liver lobule. Mild degeneration and edema of liver cells are visible, but no significant necrotic changes are observed.

Medium-dose group: Lymphocyte infiltration in the liver increases significantly. Scattered focal necrosis of varying sizes and portal inflammation are present, which are consistent with the pathological changes seen in the early stages of liver failure.

High-dose group: Diffuse hepatocyte degeneration and necrosis are visible under the microscope. Large necrotic foci are observed, consistent with the pathological diagnosis of liver failure. The liver lobule structure is disordered, with extensive hepatocyte necrosis and significant lymphocyte infiltration in the field of view.

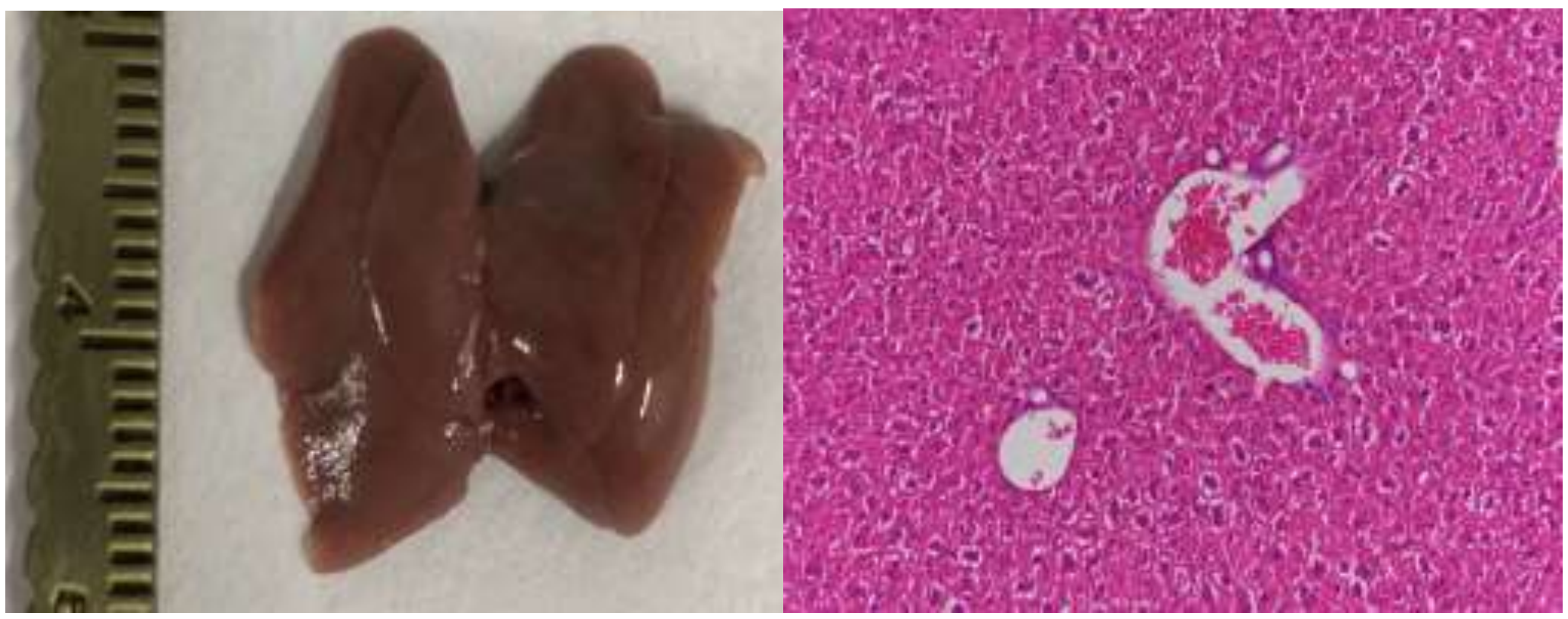

A. Control

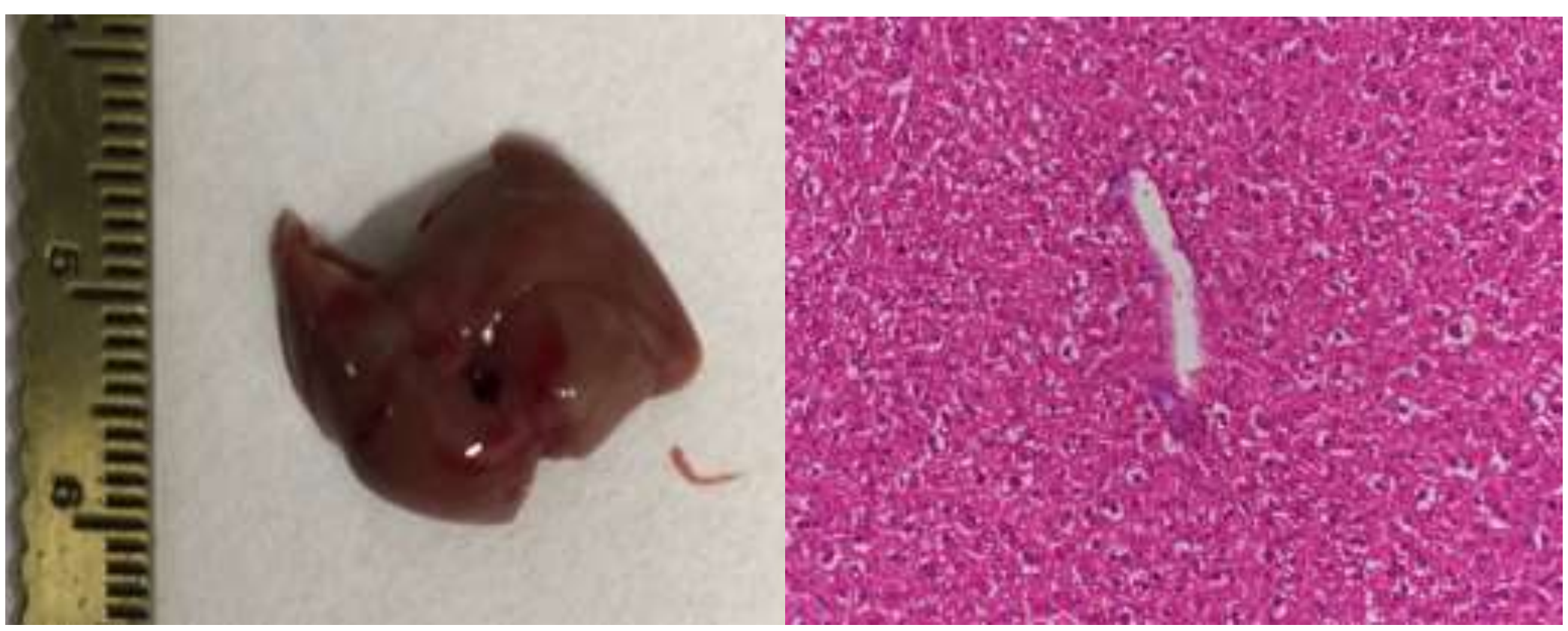

B. ConA（20μg/kg）group

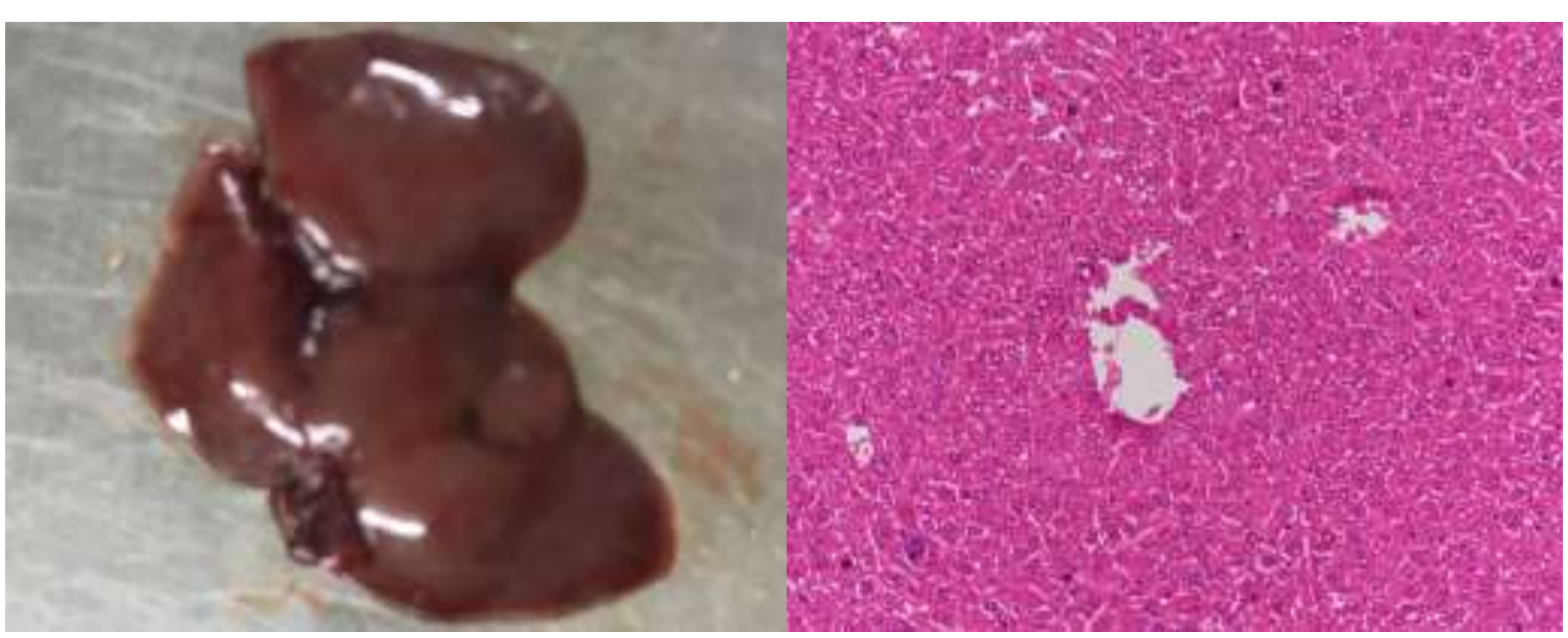

C. ConA（20μg/kg）(Model group)

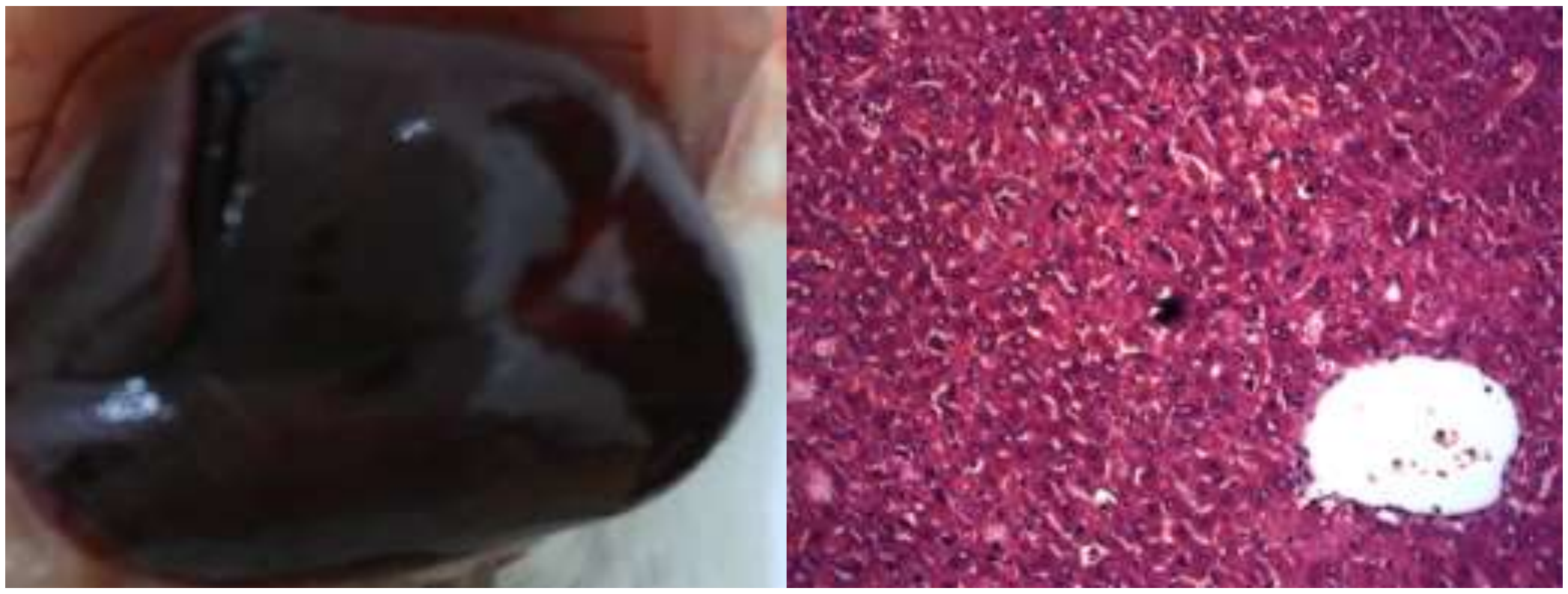

D. ConA（20μg/kg）group

Figure S3. Gross Specimens and HE Staining of Mice Livers in Various Doses of Jack Bean Protein Groups

3.2 Serum Inflammatory Cytokine Levels in Mice of Various Doses of Jack Bean Protein Groups

The serum levels of pro-inflammatory cytokines IL-4, IL-17, and TNF-α in the three model groups were significantly higher than those in the blank control group. Furthermore, as the dose of jack bean protein increased, the levels of these inflammatory cytokines also progressively increased. Combined with the pathological indices and microscopic findings of the liver tissues in each model group, it was further verified that the liver function damage, even liver failure, caused by tail vein injection of jack bean protein in mice is primarily due to immune cell infiltration and elevated levels of inflammatory cytokines, representing an immune-mediated mechanism. This mechanism is more consistent with the immune damage mechanism in liver failure and the prophase of liver failure caused by hepatitis B virus. Moreover, a medium dose of jack bean protein, specifically 20ug/kg, is considered the optimal dose for inducing liver failure in the pre-stage of mice.

Table S3. Serum Levels of IL-4, IL-17, and TNF-α in Blank Control Group and Mice of Various Doses of Jack Bean Protein Groups

Group（n=15）	IL-4（pg/mL）	IL-17((pg/mL)	TNF-α(ng/L)
Control	31.17±4.96	36.24±7.71	120.18±22.57
DonA（10ug/kg）	43.65±6.84	63.74±11.36	180.02±28.12
DonA（20ug/kg）	60.21±7.04	89.74±10.52	241.14±33.41
DonA（30ug/kg）	67.52±5.91	102.41±10.68	288.71±35.22
F	102.74*	124.96*	88.10*
